# Suitability of the global forest cover change map to assess climatic megadisturbance impacts on remote tropical forests

**DOI:** 10.1038/s41598-022-13558-7

**Published:** 2022-07-04

**Authors:** Tatiana Geler Roffe, Stéphane Couturier, Arturo García-Romero

**Affiliations:** 1 Geography Institute (Instituto de Geografía), National Autonomous University of Mexico (UNAM), Circuito Exterior s/n Ciudad Universitaria, 04510 Mexico City, Mexico; 2 Center for Complexity Science (Centro de Ciencias de la Complejidad), National Autonomous University of Mexico (UNAM), Circuito, Mario de la Cueva 20, Insurgentes Cuicuilco, Mexico City, Mexico

**Keywords:** Plant sciences, Environmental sciences, Natural hazards

## Abstract

The occurrence and combination of extreme meteorological events may lead to severe (megadisturbance) impacts on conserved forests and protected areas all over the world. Recent research has shown that megadisturbance impacts (after the events of hurricanes and prolonged drought) may be detected in subtropical forest cover using changes in spectral indices derived from satellite imagery. The objective of this study is to assess the impact of megadisturbance on forest types of the Alejandro de Humboldt National Park, Republic of Cuba in the 2001–2017 time-period. The Global Forest Cover Change (GFCC, available on the Global Forest Watch website) product was validated and indicated the prominence of megadisturbance for year 2016 (85% of the total disturbed area), largely associated with the Hurricane Matthew event. A robust estimator of the disturbed forest area, based on GFCC-stratified sampled verification sites, suggests that 11,110 ± 1,771 hectares of forest (~ 16% of the Park’s total area) was affected by megadisturbance between 2001 and 2017. In 2017, about 1276 hectares of forests were impacted, presumably related to a long-lasting effect of megadisturbance due to Hurricane Matthew and prolonged droughts in previous years. Four types of tropical rainforests (especially lowland rainforest and submountainous sclerophyllous rainforest on serpentinite), that cover 43% of the National Park, accounted for about 85% of the impacts by megadisturbance. The Easternmost portion of these forests should be prioritized for conservation monitoring and possibly for forest restoration strategies. This study contributes to establishing methodological guidelines for rapid environmental assessment of remote, tropical protected areas facing the impacts of extreme meteorological events and climate change.

## Introduction

In line with ongoing global warming, the frequence and severity of extreme meteorological events (droughts, hurricanes, tornados) is likely to increase^1,2^. Megadisturbance alludes to a critically severe environmental disturbance associated with one or the combination of several extreme meteorological events^3^. Spatial information on the extent of megadisturbance may help prevent major threats to the biodiversity and resilience of forest ecosystems^4,5^, especially with the possible advent of broad-scale wildfires after megadisturbance takes place^4,6^.

As an important step towards mitigation of ecological disasters, it is essential to assess the current impacts of extreme meteorological events on the forests of protected areas. One of the immediate effects of tropical hurricanes and tornados is the defoliation of trees^7,8^. Extreme droughts may also lead to defoliation and mortality of tree individuals, a disturbance that is detectable in forests via remote sensing tools^9,10^. Recent observations of forests in Southern states of the USA and Caribbean islands have suggested defoliation may last for several months after a tornado or hurricane event^11^ and during a prolonged drought event^12^. The combination of both events is a concern for the resilience of forest ecosystems in conservation areas in the tropical belt [Caribbean islands] and may lead to tree mortality and more severe and durable defoliation^4^.

In this research, we wish to assess impacts of megadisturbance in the Alejandro de Humboldt National Park (AHNP), among the highest forest biodiversity spots in the Republic of Cuba and in the Caribbean islands^13–15^. The Cuban archipelago has faced a series of hurricanes and prolonged drought events in the past two decades, and Hurricane Matthew (October 2016) has had a strong impact on the AHNP forests^16^. The assessment of the megadisturbance associated with these events would help enhance management plans for the conservation of fragile ecosystems and for the mitigation of impacts of potential wildfires in the protected area.

The impact of hurricanes on vegetation cover has been mapped by de Beurs et al.^12^ at 500 m spatial resolution at the country level in the Caribbean. To our knowledge, no assessment at finer resolution has been attempted in the protected areas of Cuba to explore potential impacts on forest biodiversity. A detailed forest type map circa 2011 developed by Estrada et al.^17^ provides biodiversity information nationwide at 30 m resolution, which could qualify as the highest resolution reference forest type map to study biodiversity impacts in Cuba over the past two decades.

Defoliation and impacts on vegetation after the occurrence of hurricanes and extreme droughts were assessed by de Beurs et al.^12^ using a disturbance index based on greenness, brightness and wetness spectral bands of the MODIS sensor. The algorithm of the Global Forest Cover Change (GFCC) 30 m resolution product^18^, commonly used to detect anthropic deforestation from one year to another, is based on abrupt change of several spectral vegetation indices including greenness, brightness and wetness. The "forest loss" layer of the GFCC product could therefore be tested to detect defoliation / tree mortality in years following megadisturbance such as hurricanes and/or prolonged droughts.

We propose to use the GFCC product to detect potential annual megadisturbances in AHNP between 2001 (beginning of year) and 2017 (end of year, fifteen months after the pass of Hurricane Matthew, the major tropical storm crossing the AHNP region in the past two decades), and to evaluate the impact of the megadisturbance on the forest types of the Park. As a validation for megadisturbance detection by the GFCC "forest loss" layer, we propose to assess the variation of the NDVI signal derived from satellite imagery. A major objective of our research is to explore easily reproducible methods for megadisturbance impact assessment on biodiversity for natural protected areas in the Caribbean and in the subtropical belt. The specific objectives of this study are:

1. To estimate yearly megadisturbance impact based on the GFCC "forest loss" product and an adhoc sampling of verification sites in the period 2001–2017;
2. To assess changes in NDVI value ranges against the GFCC "forest loss" product between 2001 and 2016 (end of year, 3 Months after the Hurricane Matthew event);
3. To assess the megadisturbance impact on biodiversity using the detailed forest type map of Estrada et al.^17^ for AHNP.

## Study area

**Figure 1:**
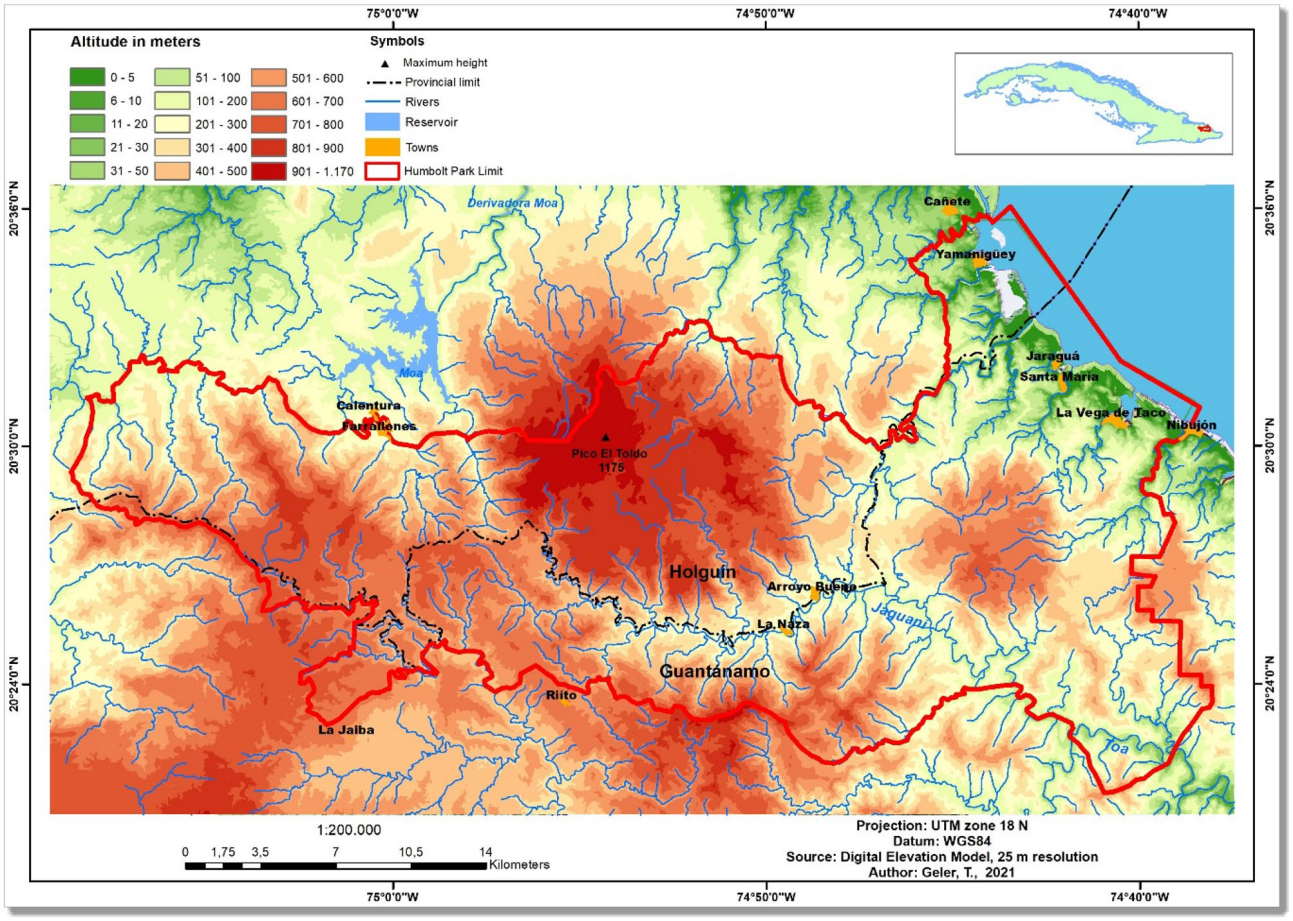
The study area: Alejandro de Humboldt National Park, Cuba. The map was created using ArcGIS 10.7 software (https://support.esri.com/en/products/desktop/arcgis-desktop/arcmap/10-7-1).

The Alejandro de Humboldt National Park (AHNP) extends over 70,680 hectares of the Holguín and Guantánamo provinces in the far Eastern coast of the Republic of Cuba (Fig. 1). AHNP was declared a world heritage site in 2001 for the quality of its natural landscapes and is the core of the Cuchilla del Toa Biosphere Reserve. In the strictly conserved area (67,000 hectares), more than 1200 animal and 1000 plant species were documented, with over 80% endemism^14^, which makes the AHNP one of the main biodiversity hotspots of the Cuban archipelago.

The demographic pressure on the Alejandro de Humboldt National Park region is low and scattered (a total of less than 5000 people in 2009^19^ living in 10 communities inside the Park and 32 communities in the vicinity of the Park). No extended wildfires were reported in the 2000–2017 time period (personal communication : Norvis Hernández Hernández). Although extraction activities may occur in the National Park, only little anthropic disturbance of the forest cover (no extended logging activity) is reported by the National Park authorities and by the World Heritage outlook assessment^14,19^. However, extended forest fires were reported after this time period, from 2018 onwards, the largest to date being in April 2021 (^19,20^; personal communication: Norvis Hernández Hernández). A perspective of this research is to contribute to the detection and protection of fragile forest ecosystems that could be more prone to fire propagation because of their past exposition to megadisturbance.

## Materials and methods

**Figure 2:**
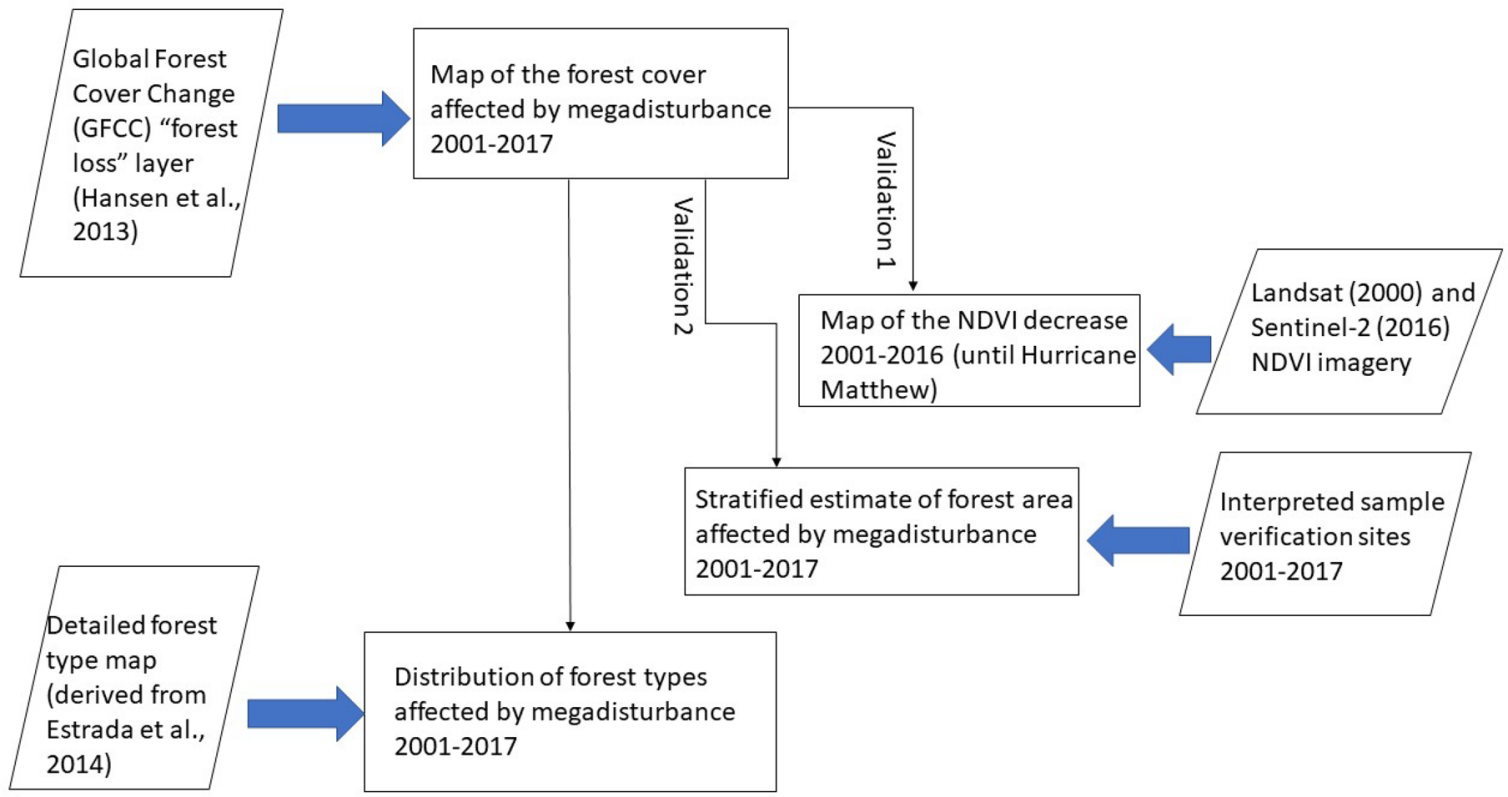
Methodological flowchart for the rapid assessment of megadisturbance impacts on protected areas with little anthropic deforestation: the case of the Alejandro de Humboldt National Park, Republic of Cuba. The Global Forest Cover Change (GFCC) product is available on the Global Forest Watch website.

In this research, we propose a methodology for the rapid assessment of megadisturbance impacts on protected areas with little anthropic deforestation. This methodology consists in four steps (Fig. 2).

In a first step, a tentative megadisturbance impact map on forests is derived from the Global Forest Cover Change product^18^ at 30 m resolution. In a second step, megadisturbance impacts are estimated per forest type using a taxonomically detailed vegetation cover map at 30 m spatial resolution. Both steps are described in the first subsection below. The third step consists in building a robust estimator of the area that corresponds to megadisturbed forest, based on the accuracy assessment of the megadisturbance impact map, as explained in sub-section "Robust estimation of the area impacted by megadisturbance in 2001–2017". The fourth and last step consists in independently estimating the area of megadisturbance impacts on forest by mapping the changes in NDVI from satellite data in the period 2001–2016 (three months after the Hurricane Matthew event). The last step provides a means of interpreting the kind of megadisturbance detected by the GFCC product and is explained under subsection "Assessment of megadisturbance impacts using NDVI analysis".

### Mapping the megadisturbance impacts on forests of the Alejandro de Humboldt National Park

The GFCC 30 m resolution product is based on a nearly complete set of imagery from the Landsat missions 4, 5, 7, and 8 over the two last decades^18^. A forest non-forest layer was established in year 2000 for the AHNP based on the "tree cover" layer of the GFCC setting a minimum threshold of 10% tree cover.

The mapping of "forest loss" events is achieved following hierarchical decision tree classifiers trained on the compared values of vegetation indices (including "greenness", "wetness" and "brightness" values) from the beginning to the end of a given year. The yearly estimate of forest loss (a “Year of gross forest cover loss event”, or “loss year” GIS layer) was downloaded with pixels encoded as 0 (no loss) or a value ranging from 1 to 17, corresponding to the occurrence of forest loss in year 2001, 2002, …, 2016, or 2017.

The anthropic disturbance in Alejandro de Humboldt National Park (AHNP) has been reported as negligible compared to the disturbance of natural hazards before 2017 (personal communication: Norvis Hernández Hernández; Hernández Rodríguez and Cruz Flores, 2016). The yearly forest loss value was therefore considered as a potential proxy for megadisturbance impacts on a yearly basis. A map of forest megadisturbance impacts was established for 2001–2017, with three classes: "forest no change"; "non-forest no change"; and "degraded forest", the latter corresponding to pixels labeled as "forest" in 2000 and "forest loss" in the period 2001–2017. Finally, taxonomically detailed vegetation cover map circa 2011 at 1:50,000 scale, including forest types at sub-community taxonomic level ("*formaciones vegetales naturales y semi-naturales de Cuba*")^21^, was established for AHNP, on the basis of the cartography developed and released freely by Estrada et al.^17^. The relative megadisturbance impact on forest communities was achieved overlaying the megadisturbance impact map with the forest type map.

### Robust estimation of the area impacted by megadisturbance in 2001–2017

On the basis of the megadisturbance impact map (see previous section), the area of the "degraded forest" class was estimated according to recommendations by Couturier et al.^22^ and Olofsson et al.^23^. A total sampling size of 751 pixels of the map was selected for verification, according to the proportional sampling size scenario in Olofsson et al.^23^. The pixels were selected according to a stratified sampling scheme for classes "forest no change", "non-forest no change" and "degraded forest"^22^. Reference labels were attributed to the verification pixels on the basis of forest cover change viewed on the high resolution imagery of the Google Earth archive in (or nearest to) year 2001 and at dates following the "forest loss" year of the GFCC product. A confidence level of interpretation was registered for each verification pixel, attributing an alternate class for low confidence cases^24^. Although validation in the field would have added confidence to the verification process, especially for the "degraded forest" class, it could not be planned in this study because of the infrastructure disturbance within months after Hurricane Matthew in the depressed region, and because the defoliation of trees, a major symptom of megadisturbance, partially fades away a few months later. Also, the GFCC product, necessary for the stratified sampling of the verification sites in forest cover change classes, is available only at the beginning of the following year.

An error matrix was built confronting the forest megadisturbance map and the reference labels. Based on the error matrix, accuracy indices, areas of land cover change classes (“forest no change”, “non-forest no change” and “degraded forest”) and corresponding 95% confidence intervals were derived from the metrics introduced by Stehman & Czaplevski^25^ for the accuracy assessment of land cover maps. Accordingly, the area of change class *k* was estimated using the stratified estimator based on our sampled reference sites (see Eq. 23 in^26^, or Eq. 9 in^23^):

1 $$\hat{A}_{k} = {\text{A*}}\mathop \sum \limits_{i = 1}^{q} W_{i} \frac{{n_{ik} }}{{n_{i.} }}$$

where *n*_*ik*_ is the sample count at cell *(i,k)* in the error matrix, *W*_*i*_ is the area proportion of class *i* on the map, and *q* is the total number of classes.

Finally, error margins at 95% probability of the areas of change classes were derived from the standard error of this estimator (see Eqs. 10 and 11 in^23^):

2 $$S\left( {\hat{A}_{k} } \right) = {\text{A*}}\sqrt {\mathop \sum \limits_{i} W_{i}^{2} \frac{{\frac{{n_{ik} }}{{n_{i.} }}\left( {1 - \frac{{n_{ik} }}{{n_{i.} }}} \right)}}{{n_{i. } - 1}} }$$

The estimation of the "degraded forest" area was derived from equations (1) and (2). The handling of pixel verification sites were carried out under the QGIS software environment.

### Assessment of megadisturbance using NDVI analysis in 2001–2016

NDVI derived from Landsat and Sentinel imagery has been used in recent studies to assess megadisturbance impacts on forests until a few months after hurricane or tornado events^8,11^. Detecting defoliation due to Hurricane Matthew using NDVI at the end of 2017 (15 months after the event) would likely not be successful because of pioneer vegetation consolidation during 15 months^12^. Therefore, we studied changes in NDVI in a time period equivalent to the 2001–2016 GFCC "forest loss" product instead of the 2001–2017 product used in previous sections. According to the GFCC "forest loss" product, around 99% of the 2001–2016 forest disturbance occurred during year 2016 (Table 1).

**Table 1:** Area of forest impacted by megadisturbance in the Alejandro de Humboldt National Park (AHNP) according to the "forest loss" layer of the Global Forest Cover Change (GFCC) product.

	Pixel count	Area (ha)	Area (% AHNP)
Total AHNP area	977,412	70,668.84	100
**Impacted forest per year**			
2001	79	5.71	0.01
2002	104	7.52	0.01
2003	38	2.75	0.00
2004	39	2.82	0.00
2005	118	8.53	0.01
2006	66	4.77	0.01
2007	48	3.47	0.00
2008	299	21.62	0.03
2009	90	6.51	0.01
2010	96	6.94	0.01
2011	72	5.21	0.01
2012	136	9.83	0.01
2013	35	2.53	0.00
2014	47	3.40	0.00
2015	87	6.29	0.01
2016	123,476	8927.56	12.63
2017	17,651	1276.20	1.81
Total impacted forest		10,301.66	14.58
Non impacted forest in 2017	834,931	60,367.18	85.42

A pair of Landsat images (1G processing level, with less than 10% cloud cover) acquired in December 2000–January 2001 and a pair of Sentinel-2 images (1C processing level, with less than 10% cloud cover) acquired in December 2016 and January 2017 were used to cover the entire AHNP park for NDVI classification. Two mosaics (circa January 2001 and circa December 2016) were computed under ENVI (for Landsat) and SNAP (for Sentinel-2) software environments. Both mosaics were converted to NDVI images and the Sentinel-2 NDVI mosaic was subsequently converted (average values) to 30 m spatial resolution imagery, comparable with the Landsat mosaic. NDVI was mapped in both epochs according to four value ranges: 0–0.25 (no vegetation); 0.25–0.50 (little vegetation cover); 0.50–0.70 (moderate vegetation cover); 0.70–1.00 (dense vegetation cover). Defoliation in Caribbean forests after hurricane events were identified by Hu and Smith^8^ as a transition from the "dense vegetation cover" value range to the "moderate vegetation cover" or lower value ranges; the area under such transition was taken as an approximation for the "degraded forest" area estimation in 2001–2016. This area was confronted with the 2001–2016 "forest loss" layer of the GFCC product. The processing of satellite imagery was carried out under the ENVI and SNAP software environments.

## Results

### Megadisturbance impacts according to the GFCC "forest loss" data

**Figure 3:**
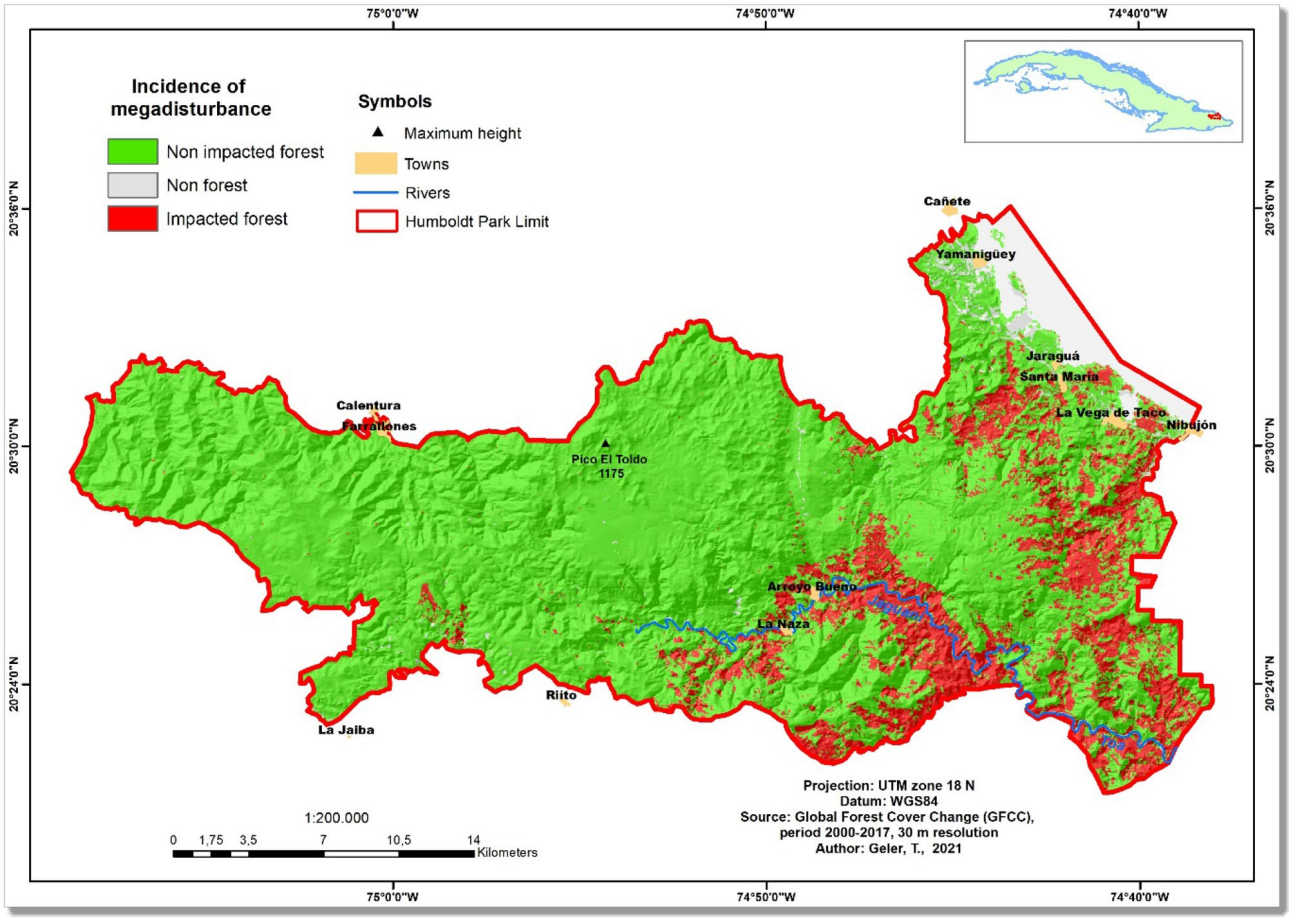
Megadisturbance map of the Alejandro de Humboldt National Park, Republic of Cuba, in the period 2001–2017, according to the Global Forest Cover Change (GFCC) product. The map was created using ArcGIS 10.7 software (https://support.esri.com/en/products/desktop/arcgis-desktop/arcmap/10-7-1).

From a total of 70,669 hectares of the Alejandro de Humboldt National Park (AHNP), 10,302 hectares were found as "degraded forests" by the end of year 2017 (Table 1), which represents about 14.6% of the total area. Most degraded forests lay near to the coast and in the watershed of the Jaguaní and Toa rivers (Fig. 3). Additional to a considerable area impacted through defoliation following the Hurricane Matthew event (8,928 hectares; 12.6% of the total National Park area), persistent defoliation was also found for a large area in year 2017 (1276 hectares; 1.8% of the total National Park area) (Table 1).

**Figure 4:**
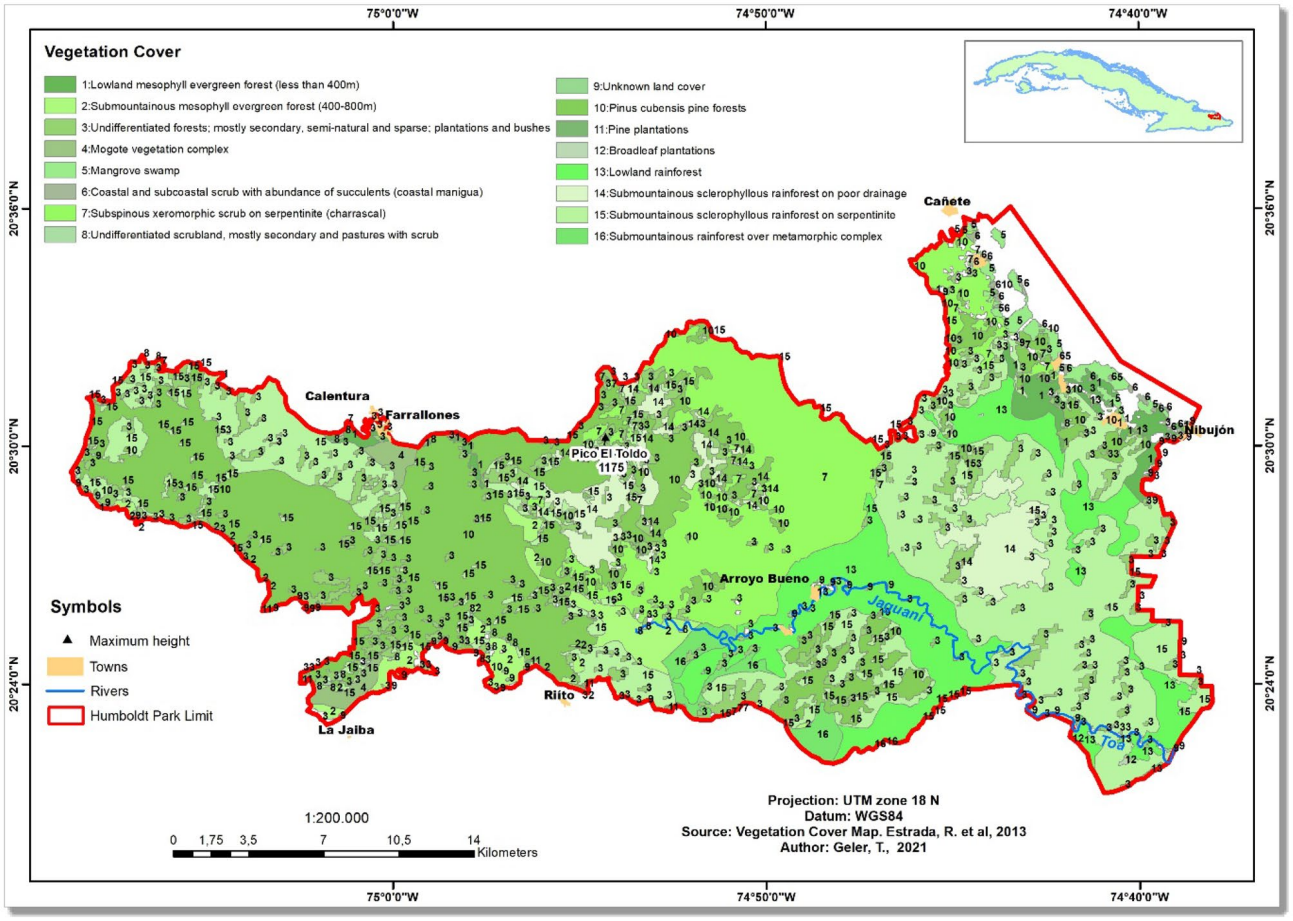
Vegetation cover map *circa* 2011 in the Alejandro de Humboldt National Park ( adapted from Estrada et al., 2013^17^). The map was created using ArcGIS 10.7 software (https://support.esri.com/en/products/desktop/arcgis-desktop/arcmap/10-7-1).

According to the vegetation cover map^17^, about 85% of the megadisturbance impact occurred in tropical rainforests (8657 hectares of *pluvisilvas*, Table 2). Lowland rainforest (*pluvisilvas de baja altitud*) and submountainous rainforest on serpentine (*pluvisilvas submontanas sobre serpentinita*), both largely occurring in the Eastern part of the National Park (Fig. 4), were the forest ecosystems with highest impact (4662 and 3288 hectares of impacted forests, respectively).

**Table 2:** Original (2000) and impacted (2001–2017) area of vegetation cover types in the Alejandro de Humboldt National Park, according to the vegetation cover map *circa* 2011 ^17^ and the GFCC "forest loss" product. The total impacted area (in hectares) is slightly different from that of Table 1 because of the difference of scale between the vegetation cover map and the GFCC product. Furthermore, some small vegetated areas in the AHNP are not included in the vegetation cover map because they were not considered natural or semi-natural vegetation. *amsl* stands for "above mean sea level".

No	Vegetation cover type	Area 2000 (ha)	%	Impacted area (ha)	%
1	Lowland mesophilic evergreen forest (less than 400 m *amsl*)	1252.29	1.86	471.97	4.63
2	Submountainous mesophilic evergreen forest (400–800 m *amsl*)	1958.58	2.91	55.99	0.55
3	Undifferentiated forests; mostly secondary, semi-natural and sparse; plantations, groves, maniguas and bushes	5474.88	8.13	256.57	2.52
4	Mogote vegetation complex	579.62	0.86	0.27	0.00
5	Mangrove swamp	703.06	1.04	91.03	0.89
6	Coastal and subcoastal scrub with abundance of succulents (coastal manigua)	138.36	0.21	21.24	0.21
7	Subspinous xeromorphic scrub on serpentinite (charrascal)	9551.78	14.19	140.23	1.38
8	Undifferentiated scrubland, mostly secondary and marabuzales, maniguas and pastures with scrub, highly degraded and sparse secondary forests	92.93	0.14	1.53	0.02
9	Unknown land cover	127.05	0.19	14.08	0.14
10	Pinus cubensis pine forests	18,139.92	26.95	468.33	4.59
11	Pine plantations	47.24	0.07	11.11	0.11
12	Broadleaf plantations	15.95	0.02	3.92	0.04
13	Lowland rainforest	7511.25	11.16	4662.41	45.74
14	Submountainous sclerophyllous rainforest on poor drainage	3209.84	4.77	228.36	2.24
15	Submountainous sclerophyllous rainforest on serpentinite	17,468.67	25.95	3287.97	32.26
16	Submountainous rainforest over metamorphic complex	1037.69	1.54	478.25	4.69
	TOTAL	67,309	100.00	10,193	100.00
	Total (Rainforests)	29,227.45	43.42	8656.99	84.93

### Total area of megadisturbance impacts according to the area estimator based on the  GFCC accuracy assessment

According to the area estimator of the GFCC product, the megadisturbance impacted 11,110 ± 1,771 hectares in the Alejandro de Humboldt National Park (about 16.5% of the 2000 forested area, see Table 3). The area derived from the “forest loss” layer of the GFCC product (10,302 hectares, Table 1) slightly underestimates this value but falls within confidence intervals of the area estimator, suggesting that the readily available GFCC product estimates persistent defoliation events with reasonable accuracy from one year to the next in AHNP.

**Table 3:** Estimated areas and accuracy indices of categories Accuracy indices, estimated areas of land cover change classes and corresponding confidence intervals, derived from our accuracy assessment of the Global Forest Cover Change (GFCC) map 2001–2017 in the Alejandro de Humboldt National Park. The areas were derived from an estimator based on the reference sites sampled in this study (see Eq. 1). The 95% confidence intervals were derived from the standard error of this estimator (see Eq. 2).

Cover change class	Area (ha)	Area (%)	95% Confidence Interval (ha)	User’s accuracy (%)	Producer’s accuracy (%)	Global accuracy (%)
Forest–no change	56,059	79.5	1747	90.4	91.7	
Non forest–no change	3340	4.7	585	78.0	78.3	
Degraded forest	11,110	15.8	1771	61.4	56.6	
Total	70,510	100				**85.6**

### Megadisturbance 2001–2016 using NDVI analysis

**Figure 5:**
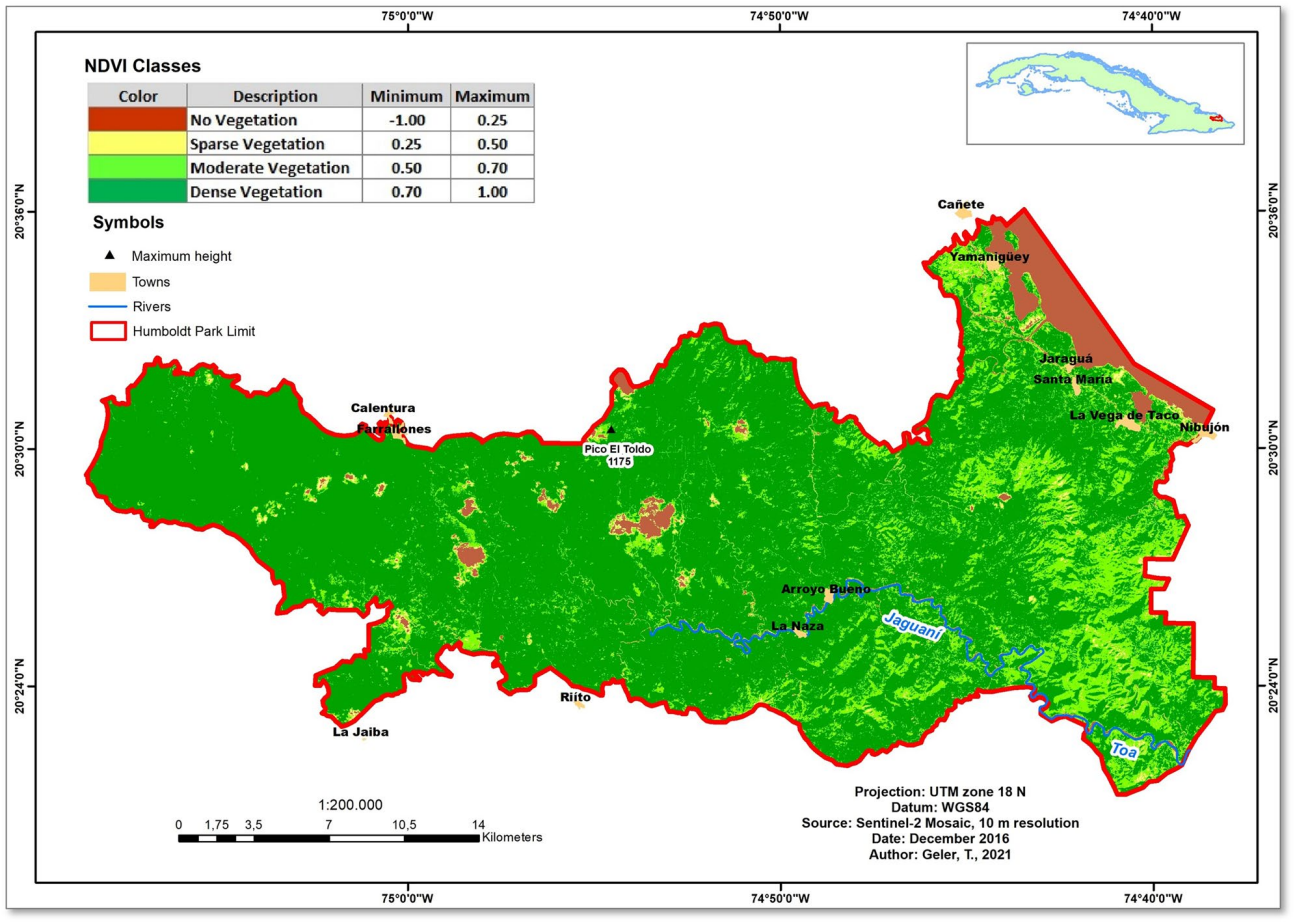
Normalized Difference Vegetation Index (NDVI) map of the Alejandro de Humboldt National Park *circa* December 2016. The map was created using ENVI 5.1 (https://www.l3harrisgeospatial.com/Support/Maintenance/ArtMID/10427/ArticleID/16174/ENVI-51), SAGAGis 8.0 (https://saga-gis.soft112.com/) and ArcGIS 10.7 (https://support.esri.com/en/products/desktop/arcgis-desktop/arcmap/10-7-1) software.

The area in the highest NDVI range (> 0.70) was 63,583.47 ha at the end of 2000 and was 55,267.19 hectares at the end of 2016 (Table 4), which suggests that 8,316.28 hectares were impacted by megadisturbance (11.8% of the AHNP). The GFCC-based megadisturbance map reported the similar value of 12.6% of the AHNP affected by megadisturbance along 2001–2016 (see Table 1). The lower values of NDVI in 2016 are distributed along the Eastern coast and in the watershed of the Jaguaní and Toa rivers (Fig. 5), similar to, although not always coincident with, the distribution of degraded forests in the megadisturbance map (Fig. 3).

**Table 4:**
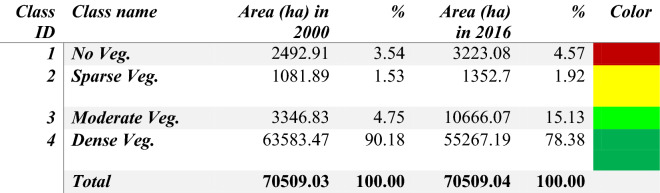
Area of the NDVI range classes *circa* December 2000 and *circa* December 2016. The color legend is the same as in Fig. 5.

## Discussion

### Temporal analysis of megadisturbance impacts in the AHNP

**Figure 6:**
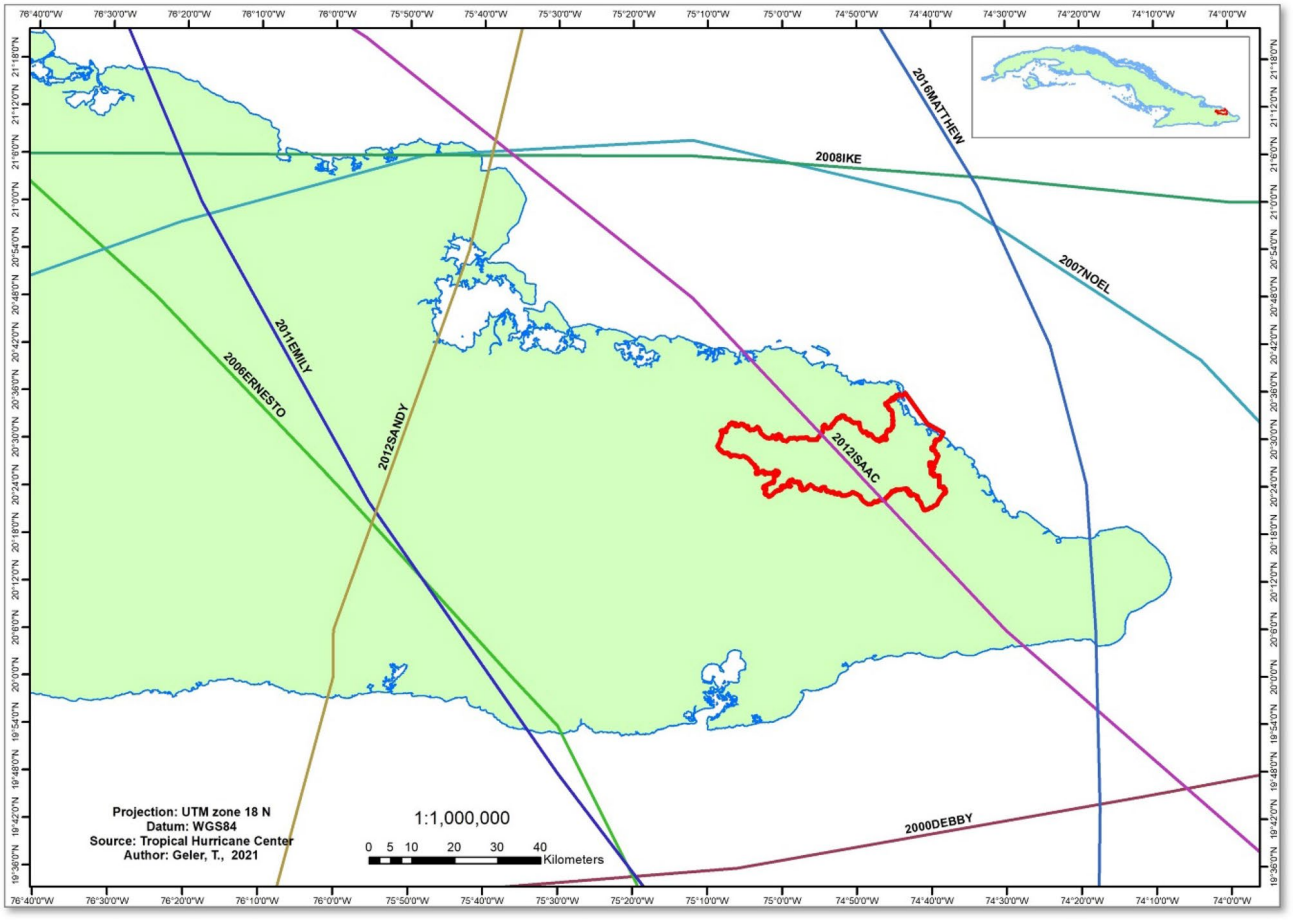
Track of the tropical storms registered in the 2000–2017 time period, easternmost region of the Republic of Cuba^27^. The Alejandro de Humboldt National Park is highlighted in red colour. The map was created using ArcGIS 10.7 software (https://support.esri.com/en/products/desktop/arcgis-desktop/arcmap/10-7-1).

Before 2016, very little impact was detected by the GFCC product (Table 1). Hernández Rodríguez and Cruz Flores^15^ likewise reported very small spectral variations between 2000 and 2010 over the National Park, suggesting little impacts on the vegetation cover. In the 2000–2015 time period, seven tropical storms were registered whose track passed through the earternmost region of Cuba (Fig. 6,^27^), including Tropical Storm Isaac (2012) whose trajectory crossed the Park. Maximum wind speed registered for Tropical Storm Isaac (2012) was about 50 knots, significantly lower than the 112 knots of Hurricane Matthew.

Two tropical storms in this region reached hurricane category in the 2000–2015 time period: Hurricane Ike (2008, 111 knots) passed about 60 km North of the National Park area, and Hurricane Sandy (2012, 100 knots) passed about 90 km West of the National Park area (Fig. 6). Wind speeds of these meteors in the National Park area were typical of tropical storms. The megadisturbance impacted area estimated for 2008 and 2012 were only 21.6 ha and 9.8 ha, respectively. A thorough visual inspection of high resolution imagery in the GoogleEarth archive confirmed this low visible impact in 2008 and 2012.

**Figure 7:**
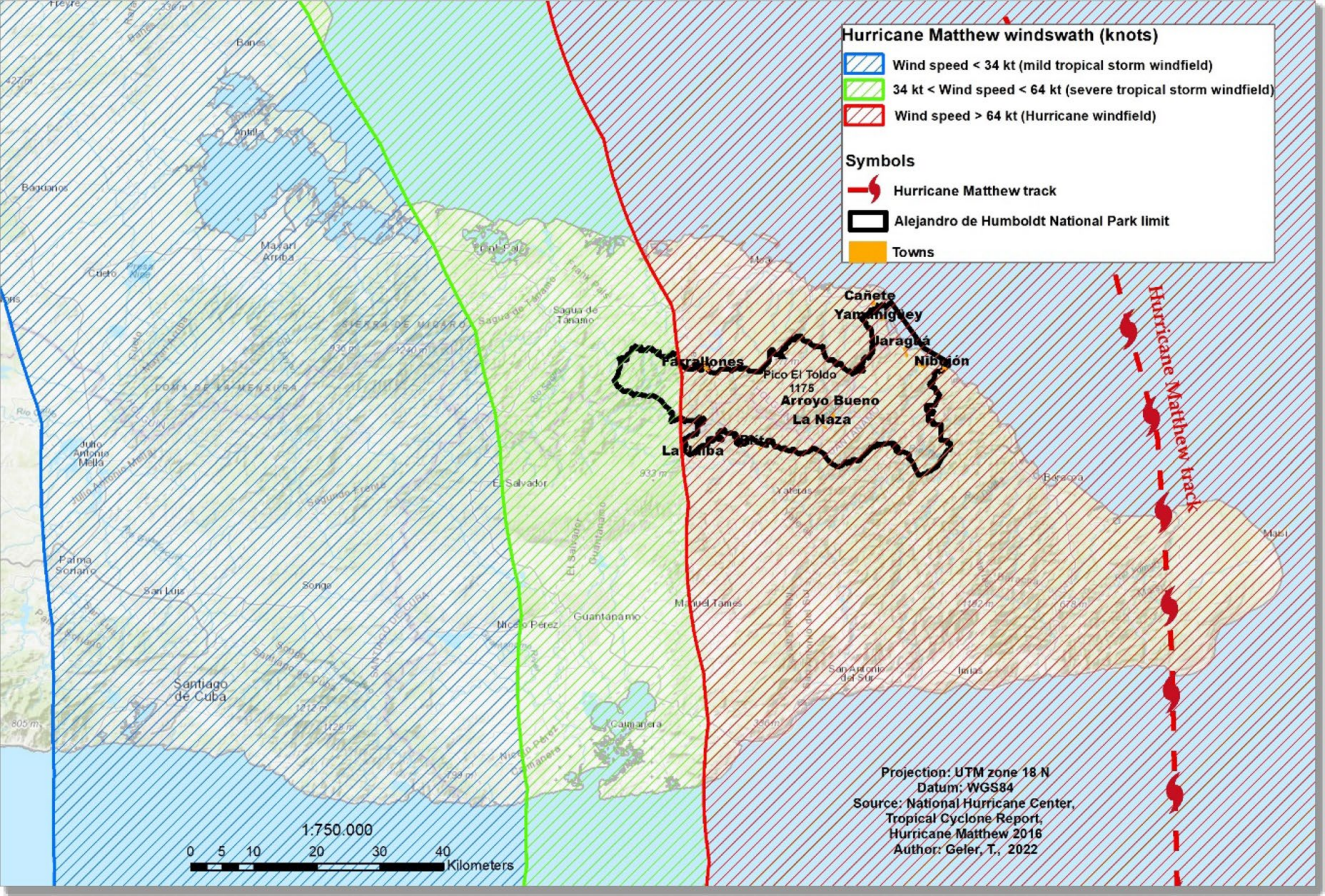
Hurricane Matthew’s track and distribution of wind fields during its passage over Eastern Cuba on 5th of October 2016^16,28^. Most of the Park area is within Hurricane force windfield. The map was created using ArcGIS 10.7 software (https://support.esri.com/en/products/desktop/arcgis-desktop/arcmap/10-7-1).

By contrast, Hurricane Matthew passed about 30 km East of the National Park area (Fig. 7,^28^), with close to maximum hurricane force wind speeds affecting the Easternmost part of the National Park.

The significative decrease in spectral indices in the AHNP in 2016–2017 with respect to 2000–2015 (Table 4) is in contrast with the general increase in NDVI found by Cruz Flores et al.^29^ accross the national protected areas between 2011–2015 and 2016–2018. The latter study was based on NDVI maps at 300 m spatial resolution, and was not meant to capture local trends. In our approach, the GFCC and NDVI products at 30 m spatial resolution provide the means of quantifying megadisturbance at the scale of National Protected Areas.

In 2016, the estimation of the area impacted using NDVI and GFCC is similar (12.6% and 11.8% of the AHNP total area, respectively). NDVI tends to capture temporary vegetation impacts whereas GFCC, based on the wetness, brightness and greenness indices, should be sensitive to longer lasting impacts^11^. This similarity suggests that megadisturbance occurred in 2016 was mainly associated with defoliation caused by the Hurricane Matthew impact^16^, remaining after three month (early October–December 2016). A depression in NDVI three month after Hurricane María was likewise reported in Puerto Rico by Hu and Smith^8^.

By contrast, in 2017, no major extreme event occurred, and yet, megadisturbance was detected in an additional 1276 hectares (1.8% of the National Park area). Perturbance, possibly related to prolonged drought, was registered in Cuban national protected areas, including AHPN, before 2016^13,30^ and in 2016^31^. The degradation detected in the "forest loss" product in 2017 could relate to a long lasting effect of the megadisturbance combining Hurricane Matthew and prolonged droughts in previous years. This interesting finding could corroborate de Beurs et al.'s hypothesis^12^ that studies based on the change detection of appropriate remote sensing spectral indices at medium resolution (10–30 m) may detect persistent defoliation and degradation following the combination of several extreme events (in this case a hurricane event and previous prolonged droughts).

### Spatial distribution of megadisturbance impacts in the forests of AHNP

Hurricane Matthew passed a few kilometers East of the Alejandro de Humboldt National Park in the Atlantic ocean (Fig. 7,^28^). The large area impacted in the easternmost part of the AHNP (especially in the Toa watershed and along the coast) seems largely related to the high wind speeds and the strength of the vortices in the vicinity of the hurricane trajectory.

**Figure 8:**
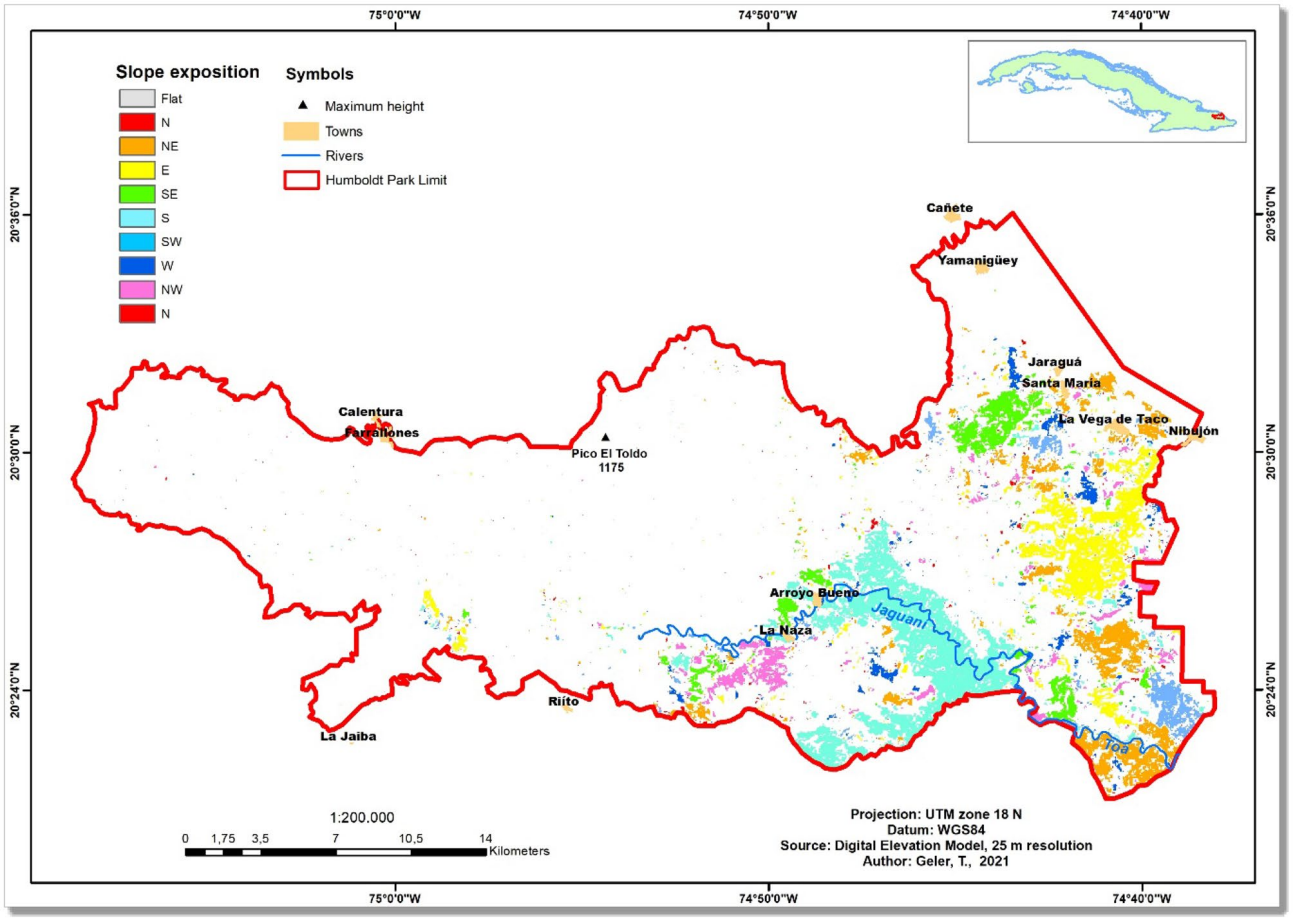
Predominant slope expositions in megadisturbance impacted forests (mapped in Fig. 3) of the Alejandro de Humboldt National Park. The map was created using ArcGIS 10.7 software (https://support.esri.com/en/products/desktop/arcgis-desktop/arcmap/10-7-1).

Additionally, according to the exposition map in Fig. 8, forests on slopes with exposition near to the South-east were most vulnerable to impacts, presumably because the general direction of Hurricane Matthew was from South-east to North-west. For example, slopes with predominant exposition to the South in the Jaguani watershed were particularly impacted (Fig. 8). By contrast, few forests with exposition to the North were impacted.

**Figure 9:**
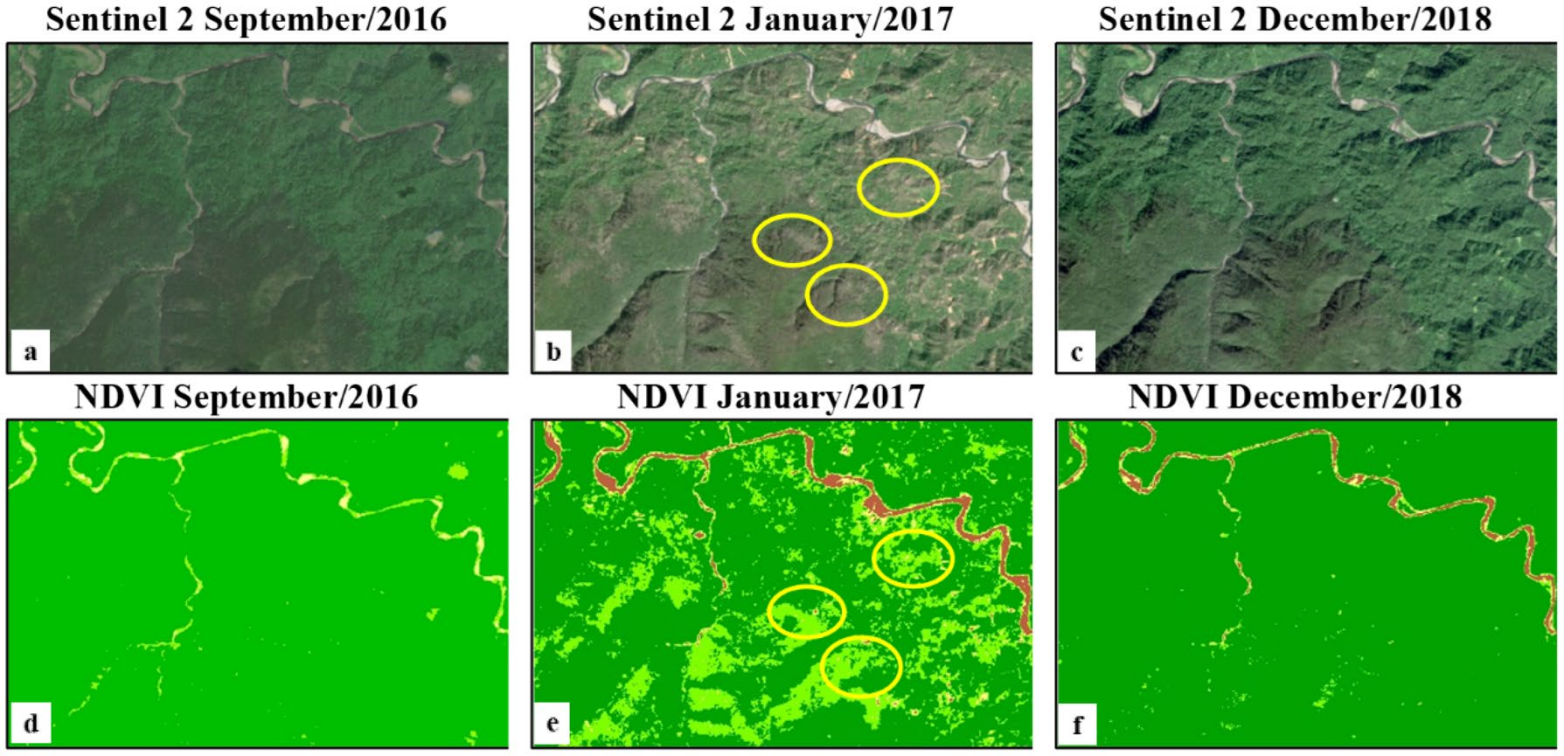
Sentinel-2 colour composites and NDVI images of the Jaguaní watershed (a tributary of the Toa river), Alejandro de Humboldt National Park, before and after the Hurricane Matthew event in October 2016.

Temporary impact on lowland rainforest areas is illustrated in Fig. 9 using Sentinel-2 colour composites and NDVI images before and after the Hurricane Matthew event. The NDVI map indicates redensification of vegetation 15 months later in these areas (Fig. 9).

**Figure 10:**
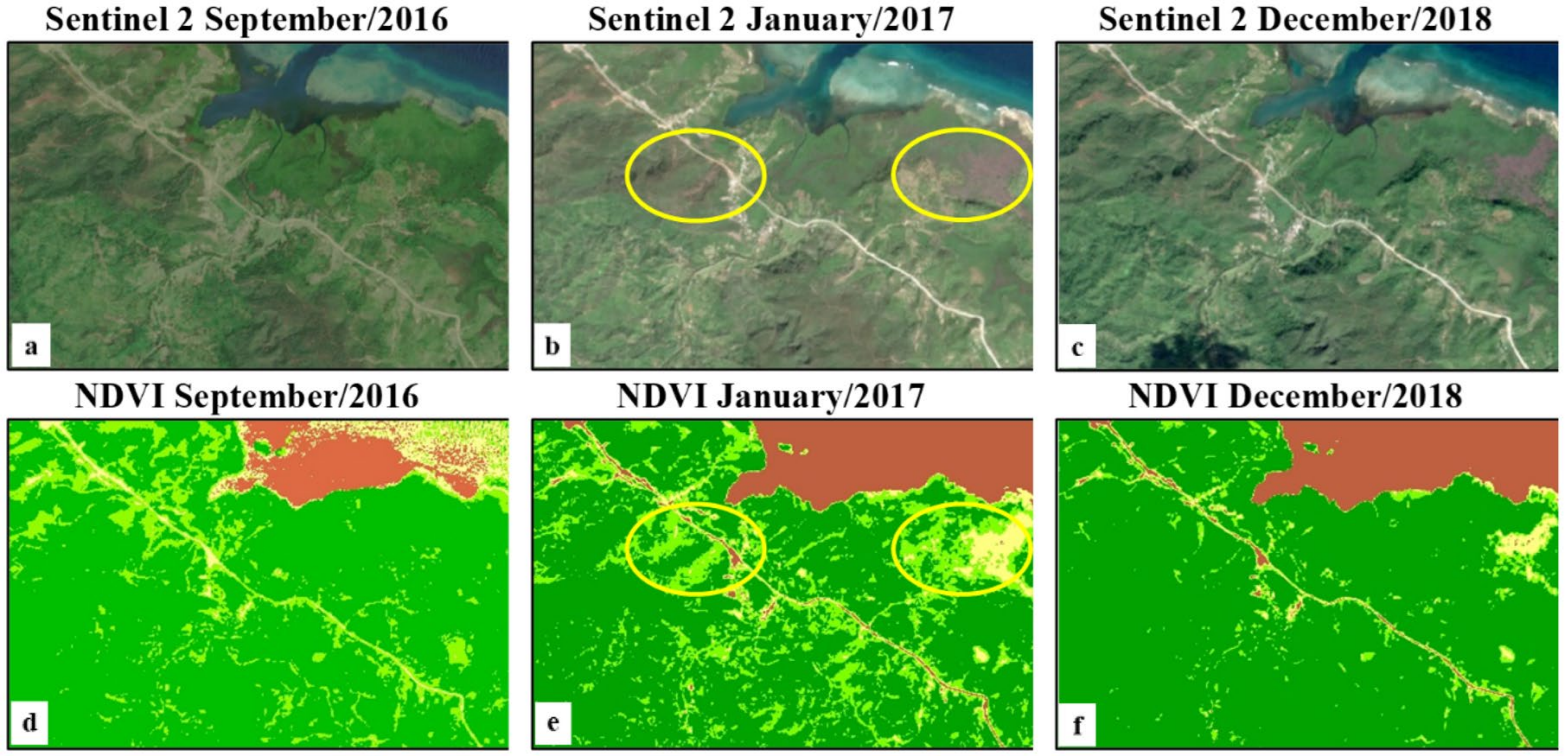
Sentinel-2 colour composites and NDVI images of a coastal area (between Taco Bay to the Southeast, and the Jaraguá Point to the Northwest), Alejandro de Humboldt National Park, before and after the Hurricane Matthew event in October 2016.

Prolonged impact on the coastal swamp forest is illustrated in Fig. 10 using Sentinel-2 colour composites and NDVI images before the Hurricane Matthew event and in December 2018 (15 Months after the event). On the right hand side of the images, persistent defoliation was observed in December 2018 in patches that showed dense vegetation in September 2016, just before the event. The local increase in sea level and the scattering of saline water during the hurricane event may both have caused high mortality of trees in parts of the coastal swamp forest.

### The combined use of the GFCC product and NDVI analysis for forest management in the face of megadisturbance events in Cuba and the Caribbean.

NDVI distribution has been statistically documented at the national level in the Republic of Cuba^12,32^ or national park level^15,29^. Additionally, remotely sensed spectral indices have been assessed in Cuba for forest loss versus forest persistence in forest management areas^33^. Based on a similar use of spectral indices, our study provides the first methodology for degradation assessment in national protected areas in the Caribbean. Our methodology and cartographic dataset could enrich the impact assessment framework of local forest management companies in charge of national protected areas in Cuba (e.g. in Baracoa for AHNP^34^).

In our study, the area impacted by megadisturbance was estimated applying good practices of area change estimation^22,23^ to the GFCC product. Accordingly, the "forest loss" layer only slightly underestimated (by 9%) the megadisturbed area (11,110 hectares). This result contrasts with results of studies on the “forest loss” layer over areas of anthropic deforestation, degradation and selective logging sites where much more underestimation was registered^35–37^. Megadisturbance events (e.g. hurricanes, prolonged droughts) may affect forests on a much larger extent and more homogeneously than anthropic intervention, which makes the GFCC “forest loss” layer more accurate at estimating impacted areas in the case of megadisturbance.

Recent degradation studies in the neotropical forests do not make use of the GFCC product to estimate degradation because most disturbance is due to shifting cultivation^38^. By contrast, in the case of little anthropic disturbance, our study suggests that the GFCC product can be useful for the assessment of megadisturbance impacts. In a long-term Typhoon study in Taiwan, Lin et al.^39^ document that it took two years for litterfall to return to pre-Typhoon levels after a major event in 1994, and annual peak leaf area index only returned to pre-event levels after ten years. This recovery timescale corroborates that the yearly forest loss GFCC product could successfully capture the spatial distribution of megadisturbance impacts in subtropical settings. According to de Beurs et al.^12^, recovery from megadisturbance appeared much slower using the disturbance index (DI), than using NDVI. The GFCC "forest loss" product is partly based on the greenness, wetness and brightness indices of the Landsat sensor bands, which are used to compute the DI index.

Limitations of our study include difficulties in the accuracy assessment process: Verification sites, visible on the high resolution imagery of the Google Earth archive, are not necessarily identifiable in the Landsat imagery used to generate the GFCC "forest loss" product. As a consequence, in mountainous settings, geometric errors due to cumulated uncertainties in the georeferenciation of the Landsat imagery and of the high resolution imagery could generate errors in the accuracy assessment process. This difficulty is hard to overcome with the visual assessment of sites in some homogenous forested environments on the Landsat imagery because of the (too coarse) 30 m spatial resolution. Annual forest loss maps at 10 m resolution (near to crown scale) derived from Sentinel-2, for example, should be more adapted to the application of forest degradation estimation in the future.

## Conclusion

The megadisturbance impact of Hurricane Matthew (2016) on forests was estimated to affect 8,928 hectares of the Alejandro de Humboldt National Park (equating 12.0% of the Park). Additionally, megadisturbance impacts were detected on 1276 hectares of forests (1.8% of the Park) in 2017, presumably a long lasting effect of Hurricane Matthew combined with prolonged droughts that were reported in previous years and in 2016. Four major types of tropical rainforests (with a total extension over 43% of the Park’s area, especially lowland rainforest and submountainous sclerophyllous rainforest on serpentinite), received 85% of the impacts by megadisturbance. Patches of persistent defoliation in December 2017 (15 Months after the Hurricane Matthew event) are of particular importance for future studies on forest resilience after megadisturbance.

Our study supports the view that the GFCC product (available for free on the Global Forest Watch website) is adequate to study annual megadisturbance in nearly uninhabited natural protected areas in the tropics. The area estimation derived from the accuracy assessment in our study suggests that the GFCC product is likely more suitable for the estimation of megadisturbance impacted areas than for the estimation of areas related to anthropic disturbance, because of the strong underestimation in the latter case, as documented in the literature elsewhere.

Our study provides the first methodology for degradation assessment in protected areas in Cuba, including a 30 m resolution map of forests impacted by megadisturbance associated with Hurricane Matthew, which could stand as a cartographic baseline for forest resilience studies in the Alejandro de Humboldt National Park. Furthermore, in the context of global environmental change and the increasing frequency of extreme meteorological events, this approach could be applied to assess megadisturbance over natural protected areas in the subtropical belt.

## Supplementary Information

Supplementary Information.

## Data Availability

The primary data underlying this research are from free access space agency repositories (Sentinel-2 and Landsat imagery) and from the free access Global Forest Watch website. In the context of accelerating environmental change and extreme meteorological events, all data generated in our study is made available to ease rapid assessments in other tropical forest settings.
