# Supplementary material for: Suitability of the global forest cover change map to assess climatic megadisturbance impacts on remote tropical forests
Source: Sci Rep. 2022 Jul 4;12:11249. doi: 10.1038/s41598-022-13558-7 (PMC9253124; doi:10.1038/s41598-022-13558-7)
Supplement: Supplementary file 1 — Supplementary Information. [file 41598_2022_13558_MOESM1_ESM.docx]

**Annex 1**: Vegetation cover types in the Alejandro de Humboldt National Park (adapted from Estrada et al. [17]) and equivalent terms in English.

| **No.** | **Tipo de cobertura vegetal** | **Vegetation cover type** |
| --- | --- | --- |
| 1 | *Bosque siempreverde mesófilo de baja altitud (menor de 400m)* | Lowland mesophilic evergreen forest (less than 400m) |
| 2 | *Bosque siempreverde mesófilo submontano (400-800m)* | Submountainous mesophilic evergreen forest (400-800m) |
| 3 | *Bosques indiferenciados; mayoritariamente secundarios, seminaturales y ralos; plantaciones, arboledas, maniguas y matorrales* | Undifferentiated forests; mostly secondary, semi-natural and sparse; plantations, groves, maniguas and bushes |
| 4 | *Complejo de vegetación de mogote* | Mogote vegetation complex |
| 5 | *Manglar* | Mangrove swamp |
| 6 | *Matorral costero y subcostero con abundancia de suculentas (manigua costera)* | Coastal and subcoastal scrub with abundance of succulents (coastal manigua) |
| 7 | *Matorral xeromorfo subespinoso sobre serpentinita (charrascal)* | Subspinous xeromorphic scrub on serpentinite (charrascal) |
| 8 | *Matorrales indiferenciados, mayoritariamente secundarios y marabuzales, maniguas y pastos con matorrales, bosques secundarios muy degradados y ralos* | Undifferentiated scrubland, mostly secondary and marabuzales, maniguas and pastures with scrub, highly degraded and sparse secondary forests |
| 9 | *Cobertura terrestre desconocida* | Unknown land cover |
| 10 | *Pinares de Pinus cubensis* | Pinus cubensis pine forests |
| 11 | *Plantaciones de Pino* | Pine plantations |
| 12 | *Plantaciones latifolias* | Broadleaf plantations |
| 13 | *Pluvisilva de baja altitud* | Lowland rainforest |
| 14 | *Pluvisilva esclerófila submontana sobre mal drenaje* | Submountainous sclerophyllous rainforest on poor drainage |
| 15 | *Pluvisilva esclerófila submontana sobre serpentinita* | Submountainous sclerophyllous rainforest on serpentinite |
| 16 | *Pluvisilva submontana sobre complejo metamórfico* | Submountainous rainforest over metamorphic complex |
